# Associations of physical activity with sarcopenia and sarcopenic obesity in middle-aged and older adults: the Louisiana osteoporosis study

**DOI:** 10.1186/s12889-022-13288-5

**Published:** 2022-05-05

**Authors:** Yan Du, Tao Xu, Zenong Yin, Sara Espinoza, Yiqiong Xie, Caleb Gentry, Qing Tian, Lan-Juan Zhao, Hui Shen, Zhe Luo, Hong-Wen Deng

**Affiliations:** 1 School of Nursing, University of Texas Health Science Center at San Antonio, San Antonio, Texas USA; 2 College of Public Health, University of Iowa, Iowa City, Iowa, USA; 3 Department of Public Health, University of Texas at San Antonio, San Antonio, Texas USA; 4 San Antonio Geriatric Research, Education, and Clinical Center, San Antonio, Texas USA; 5 Division of Geriatrics, Gerontology & Palliative Medicine, University of Texas Health Science Center at San Antonio, San Antonio, Texas USA; 6 Sam and Ann Barshop Institute for Longevity and Aging Studies, University of Texas Health Science Center at San Antonio, San Antonio, Texas USA; 7 Real World Research, Ontada, The Woodlands, Houston, USA; 8 Brown School at Washington University in St. Louis, St. Louis, USA; 9 Center for Bioinformatics and Genomics, School of Medicine, Tulane University; New Orleans, LA, New Orleans, United States of America

**Keywords:** Physical activity, Exercise, Obesity, Sarcopenia, Sarcopenic obesity

## Abstract

**Background:**

This study examined the associations between physical activity, obesity, and sarcopenia in middle-aged and older adults.

**Methods:**

We analyzed the data of 8, 919 study participants aged between 45 to 97 (mean age = 57.2 ± 8.8) from a Southern state in the United States. Self-reported physical activity was classified to regular exercise ≥ 3 times/week, < 3 times/week, and no regular exercise. Associations between physical activity, obesity and sarcopenia were explored with generalized linear models and ordinal logistic regressions stratified by age (middle-aged and older adults) and gender adjusting for covariates.

**Results:**

In middle-aged and older adults, all examined obesity related traits (e.g., body mass index, waist circumference) were inversely associated with physical activity levels (*p* < 0.01) in both genders. Exercising ≥ 3 times/week was negatively associated with lean mass indicators (e.g., appendicular lean mass) in middle-aged and older females (*p* < 0.01), while the negative associations become positive after adjusting for weight. Positive associations between physical activity and grip strength were only found in middle-aged males (*p* < 0.05). Ordinal logistic regression revealed that those exercising ≥ 3 times/week were less likely to have obesity, sarcopenia, and sarcopenia obesity in all groups (*p* < 0.01), except for sarcopenia in older males and females (*p* > 0.05). Positive associations of exercising < 3 times/week with sarcopenia and sarcopenia obesity were only found in middled adults.

**Conclusion:**

The associations of exercise frequency with obesity and sarcopenia vary considerably across gender and age groups. Exercise programs need to be individualized to optimize health benefits. Future research exploring physical activity strategies to balance weight reduction and lean mass maintaining is warranted in middle-aged and especially older adults.

## Background

During the ageing process, changes in body composition such as a progressive decline in muscle mass and strength and increase in adiposity, are observed starting as early as 30’s [1–4]. These changes predispose individuals to an increased risk of obesity, sarcopenia (loss of skeletal muscle mass and strength and physical performance), and sarcopenic obesity, which are related to a variety of cardiometabolic conditions, frailty, disability, and decreased quality of life [5–8].

Engaging in physical activity is beneficial to various health outcomes, including healthier body composition [9, 10]. The inverse associations of physical activity and obesity measurements, such as body mass index (BMI), waist circumference, and body fat percentage have been evidenced in both observation and intervention studies in adults of different age groups and in both genders [11–14].

Despite both observational and interventional studies report high levels of physical activity contribute to increased physical fitness and decreased body fat, little or no effect was found on lean body mass in older age groups [15–17]. In addition to lean body mass, the effects of physical activity on other sarcopenia related measurements, such as muscle strength, is also inconclusive. For example, a meta-analysis of 24 trials in older adults found that significant results of physical activity on grip strength were not reported for all exercise approaches, and recommended that implementing increased physical activity focused on task-specific procedures can increase grip strength [18]. In addition, a recent review reported that there is limited evidence of any effect of physical activity on sarcopenia based on clinical trials and prospectus studies, but promising results were found from observational studies [19]. Another systematic study reviewed 14 cross-sectional studies reported that in general there is positive association between physical activity and sarcopenia in older adults [20]. However, most studies included in the review assessed the relationship between physical activity and sarcopenia related traits but didn’t classify sarcopenia; or they examined older populations in Asian or European countries [20–24]. Some recent studies were also conducted in Asian or European countries [25–27], but rarely in the United States.

Additionally, despite sarcopenic obesity is increasingly prevalent in older adults, studies of physical activity on sarcopenic obesity are limited in older adults and the results are conflicting [27]. Furthermore, muscle mass decline starts around 30’s with 3–8% muscle loss during the first decline decade, and middle-aged adults are also at high risk of having sarcopenia [28, 29]. However, the study of sarcopenia and sarcopenic obesity, as well as their associations with physical activity in middle-aged adults are scarce. Therefore, using a large sample size, this study aimed to examine the relationships between exercise frequency with obesity, sarcopenia, and sarcopenic obesity in middle-aged and older adults stratified by gender.

## Methods

### Study population

The Louisiana Osteoporosis Study (LOS) is an ongoing cross-sectional study with beginning recruitment in 2011. It was designed to build a large sample pool (with ~ 20,000 subjects) and database for investigating genetic and environmental risk and protective factors for osteoporosis, obesity, sarcopenia, and other complex diseases. Subjects aged 18 and over were recruited in New Orleans, Baton Rouge, and surrounding areas in Louisiana. The detailed inclusion and exclusion criteria can be found in previous publications [30, 31]. We analyzed data for 8, 919 participants from the 2011–2020 data sets who had complete survey information and valid anthropometric and scan measurements. Consent forms were obtained from each participant before any data collection. The study was approved by the Tulane University Institutional Review Board.

### Measurements

#### Body composition, anthropometrics, and grip strength

The dual-energy X-ray absorptiometry (DXA) machine (Hologic Inc., Bedford, MA, USA) provided the measurements of body composition: whole body fat mass, whole body lean mass, percent of fat mass (% fat mass), and appendicular skeletal lean mass. The DXA machine was calibrated daily, and software and hardware were kept up to date during the data collection process. Fasting was not required in participants before taking the DXA scans. Height and weight were measured in light, indoor clothing and without shoes using a calibrated stadiometer and a calibrated balance beam scale, respectively. Body mass index was calculated as weight divided by height squared (kg/m2). The sum of the non-fat mass and non-bone of the four limbs was used to determine the appendicular skeletal lean mass (ASM), and skeletal muscle index (SMI) is calculated as ASM/height^2^ [32]. Grip strength of both hands was measured in duplicate by a hand-held dynamometer (TEC Inc., Clifton, NJ) with the maximum value recorded in kilograms. In this study, obesity-related traits were assessed by BMI, body weight, whole body fat mass, and % fat mass; sarcopenia-related traits were assessed by whole body lean mass, skeletal muscle index (SMI), and grip strength [8, 32].

#### Body composition status

Body composition status was categorized into obesity, sarcopenia, sarcopenic obesity, and normal body composition. According to previous work [27], obesity was defined by meeting at least one of the following three criteria: 1) BMI ≥ 30; 2) increased % fat mass (> 42 for females and > 30 for males; or 3) high waist circumference (≥ 88 for females and ≥ 102 for males). Using the 2018 definition set by the European Working Group on Sarcopenia in Older People (EWGSOP2) [33], sarcopenia referred as low muscle mass (SMI < 5.5 for females and SMI < 7 for males) in combination with reduced grip strength (< 16 kg for females and < 27 kg for males). Those with sarcopenia and concurrent obesity were considered having sarcopenic obesity [27]. Normal body composition was defined as no presence of any obesity, sarcopenia or sarcopenic obesity conditions.

#### Physical activity

Physical activity was measured using responses to the questions, “Do you currently exercise on a fairly regular basis?” and “How many times do you exercise per week?” Self-reported exercise frequency was categorized into three levels: exercising 3 or more times per week, exercising 1 to 2 times per week, and self-reported no regular exercise. The physical activity measures have been used in several published scientifical articles [8, 34]. According to the Physical Activity Guidelines for Americans 2nd edition [10], participating in physical activity at least 3 days per week is associated with significant health benefits and even participating in smaller amount of physical activity (e.g., 1 to 2 times a week) is associated with health benefits compared to no physical activity [35, 36]. Physical activity is any body movement that increases energy expenditure; exercise is a subcategory of physical activity which is planned, structured, repetitive, and focusing on improvement or maintenance of physical fitness [37]. To be noted, in this study, we only assessed exercise frequency.

#### Demographics and other covariates

Questionnaire survey was used to collect data including age, gender, race/ethnicity, menopausal status, and other behavioral factors which are reported to be associated with body composition indicators [38–41]. Self-reported race/ethnicity was identified by choosing from the following options: African American/Black, Asian, Caucasian/White, Hispanic/Latino, Native American/Pacific Islander and other. An affirmative response to the question “Are you postmenopausal?” was considered as post-menopause. Other behavioral factors assessed by yes/no responses to questions regarding smoking status, alcohol consumption, milk consumption (including fortified soy, rice, and almond mink), calcium supplement intake, and whether the participant had an average of 15 min of daily sun exposure. According to muscle declining significantly around one decade starting 30’s, as well as the classification of previous study and the age-specific medical subject heading, age in years was categorized into middle-aged group (45 to 64 years old) and older group (65 and over) [28, 42].

### Statistical analysis

Continuous and categorical variables were reported as mean ± std and number (percentage). ANOVA and Chi-square tests were applied to test differences in demographics, body composition, grip strength, behavioral factors by physical activity levels and gender. Missing values and outliers were cleaned. Generalized linear models were performed to assess adjusted (age, race/ethnicity, plus postmenopausal status in females) mean differences in obesity and sarcopenia related traits across physical activity levels (no regular exercise, < 3 times/week and ≥ times/week) and age groups (45–64, and 65 ~ years old) [42].

Generalized linear models were adopted to assess the associations of physical activity with obesity- and sarcopenia-related traits in the two age groups using participants who did not engage in regular physical activity as the reference group. We adjusted for age in years, race/ethnicity, smoking status, alcohol drinking, sun exposure, milk consumption, and calcium supplement intake. We also adjusted for post-menopausal status in females. For sarcopenia-related traits, in addition to those covariates adjusted for obesity related traits (model 1 s), we also adjusted for body weight because weight loss may be the result of a decrease in lean mass, especially in older adults [16, 43, 44]. Normality was checked and assured. Multicollinearity was checked using Variance Inflation Factor (VIF). The VIFs for each included variables in the models were less than 1.20. Johnston and colleagues reported that a VIF of 2.5 or even 10, is an indicator of considerable collinearity [45]. Therefore, we determined that no independent variables included in the models were highly correlated.

In addition, we used ordinal logistic regression to assess the relationship between physical activity levels with obesity, sarcopenia and sarcopenic obesity by age and gender groups, respectively. For each logistic regression model, we adjusted for age in years, race/ethnicity, smoking status, alcohol drinking, sun exposure, milk consumption, and calcium supplement intake, plus post menopause in females. We didn’t adjust for body weight in logistic regression models as we did in the generalized linear models because body weight was accounted in the body composition status categories. Self-reported no regular exercise served as the reference group for physical activity levels, and normal body composition as the reference group for body composition status. All the statistical analyses were performed with SAS software, version 9.4. A *p* < 0.05 was considered statistically significant.

## Results

In the present study (*n* = 8, 919), older adults made around 18.17% of the study population. Older males had the highest proportions of engaging in ≥ 3 times/week regular exercise (64.10%; Table 1). Non-Hispanic whites tended to have a higher proportion of people engaging in regular weekly exercise in both genders. Hispanics had the highest proportion of people who reported not exercising regularly in both males (34.41%) and females (42.61%). Hispanic females (44.32%) and non-Hispanic Asian males (53.13%) were least likely to engage in regular exercise three or more times per week. In both genders, compared to those who reported engagement in regular exercise, those who did not engage in regular exercise had the lowest proportions of people reported calcium supplement intake (32.47% in females; 5.82% in males) and milk consumption (70.56% in females; 78.44% in males). One the contrary, the highest proportions of people reported daily sun exposure were found in those exercised ≥ 3 times/week (71.02% in females; 80.22% in males).

**Table 1:** Characteristics of the sample by physical activity levels and gender

	**Regular exercise-No**	**Regular exercise- < 3/week**	**Regular exercise ≥ 3/week**	**P**
**Female (*n* = 5135)**	**(*n* = 1814)**	**(*n* = 555)**	**(*n* = 2766)**	
Age group (n, %)				0.19
45–64 years old	1440 (35.69)	447 (11.08)	2148 (53.23)	
≥ 65 years old	374 (34.00)	108 (9.82)	618 (56.18)	
Postmenopausal (female, n, %)	1274 (70.23)	380 (68.47)	1941 (70.17)	0.70
Race/ethnicity				< .001
Non-Hispanic White	1092 (32.91)	330 (9.95)	1896 (57.14)	
Non-Hispanic AA	534 (41.46)	162 (12.58)	592 (45.96)	
Non-Hispanic Asian	113 (32.01)	40 (11.33)	200 (56.66)	
Hispanics	75 (42.61)	23 (13.07)	78 (44.32)	
Health behaviors (n, %)				
Calcium supplement (yes)	589 (32.47)	243 (43.78)	1261 (45.59)	< .001
Smoke (yes)	771 (42.50)	209 (37.66)	1095 (39.59)	0.05
Alcohol drinking (yes)	1077 (60.03)	392 (71.40)	1939 (70.61)	< .001
Milk consumption (yes)	1280 (70.56)	432 (77.84)	2066 (74.69)	< .001
Sun exposure (yes)	1227 (67.64)	417 (75.14)	2219 (80.22)	< .001
Height (m)	1.61 ± 0.07	1.62 ± 0.07	1.62 ± 0.06	< .001
Weight (kg)	77.63 ± 19.46	73.30 ± 17.89	70.29 ± 17.33	< .001
	**Regular exercise-No**	**Regular exercise- < 3/week**	**Regular exercise- ≥ 3/week**	**P**
**Male (*n* = 3784)**	**(*n* = **1169**)**	**(*n* = **372**)**	**(*n* = **2243**)**	
Age group (n, %)				0.03
45–64 years old	1019 (31.33)	331 (10.18)	1902 (58.49)	
≥ 65 years old	150 (28.20)	41 (7.71)	341 (64.10)	
Race/ethnicity				< .001
Non-Hispanic White	457 (28.02)	122 (7.48)	1052 (64.50)	
Non-Hispanic AA	618 (33.08)	215 (11.51)	1035 (55.41)	
Non-Hispanic Asian	62 (32.29)	28 (14.58)	102 (53.13)	
Hispanics	32 (34.41)	7 (7.53)	54 (58.06)	
Healthy behaviors (n, %)				
Calcium supplement (yes)	68 (5.82)	42 (11.29)	191 (8.52)	< .001
Smoke (yes)	810 (69.29)	262 (70.43)	1526 (68.03)	0.56
Alcohol drinking (yes)	796 (69.46)	258 (70.11)	1608 (72.50)	0.16
Milk consumption (yes)	917 (78.44)	307 (82.53)	1849 (82.43)	< .01
Sun exposure (yes)	967 (82.72)	322 (86.56)	2036 (90.77)	< .001
Height (m)	1.74 ± 0.07	1.74 ± 0.08	1.74 ± 0.07	0.49
Weight (kg)	84.92 ± 19.16	83.17 ± 19.20	81.92 ± 16.55	< .001

Note: *AA* = African American; Sun exposure = at least 15 min sun exposure a day

### Physical activity and obesity related traits

**Fig. 1:**
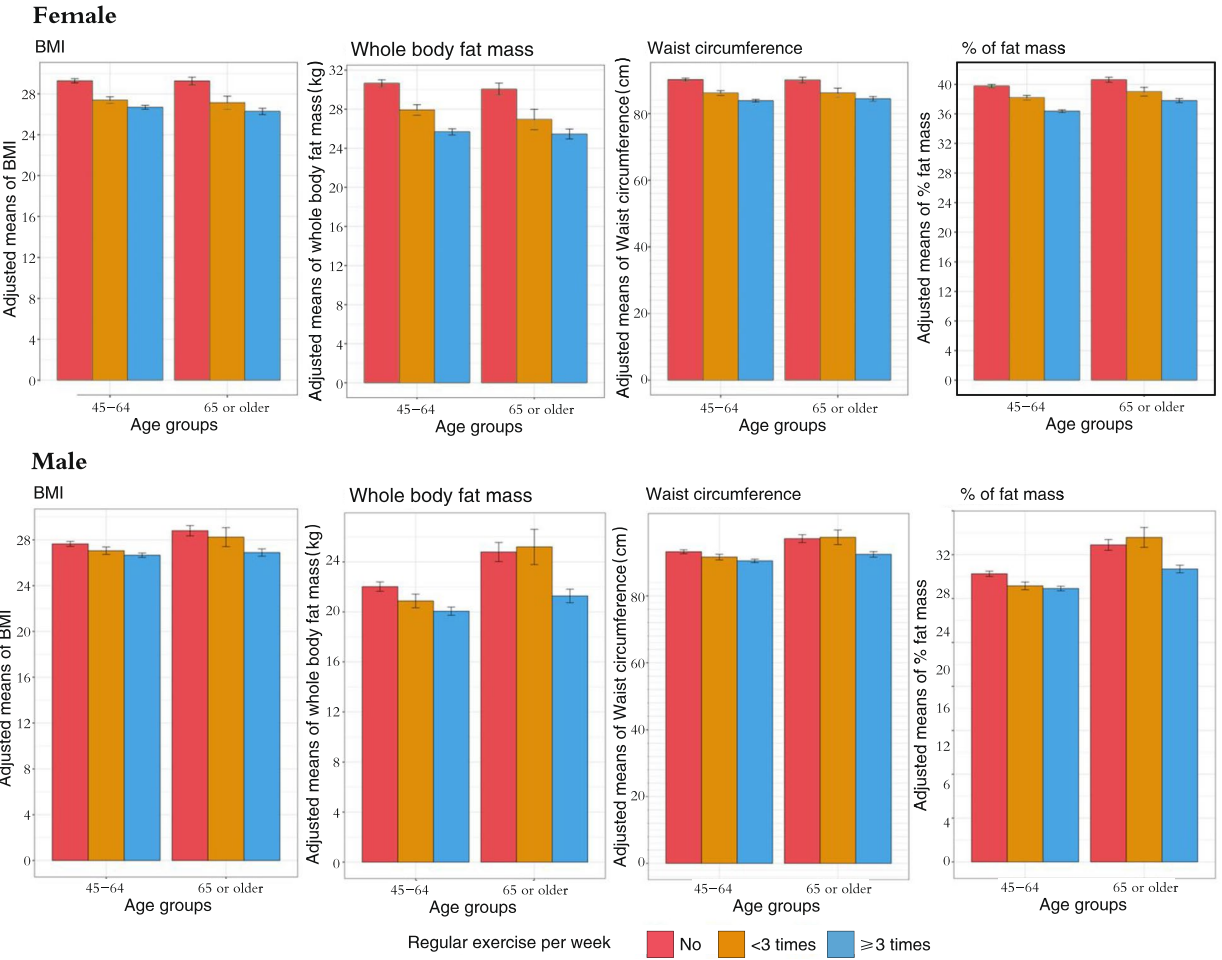
Adjusted means (standard error) of obesity across exercise and age groups. Age in years and race/ethnicity; plus menopause status for females were adjusted. BMI = body mass index. *P* < 0.001 for all traits

**Fig. 2:**
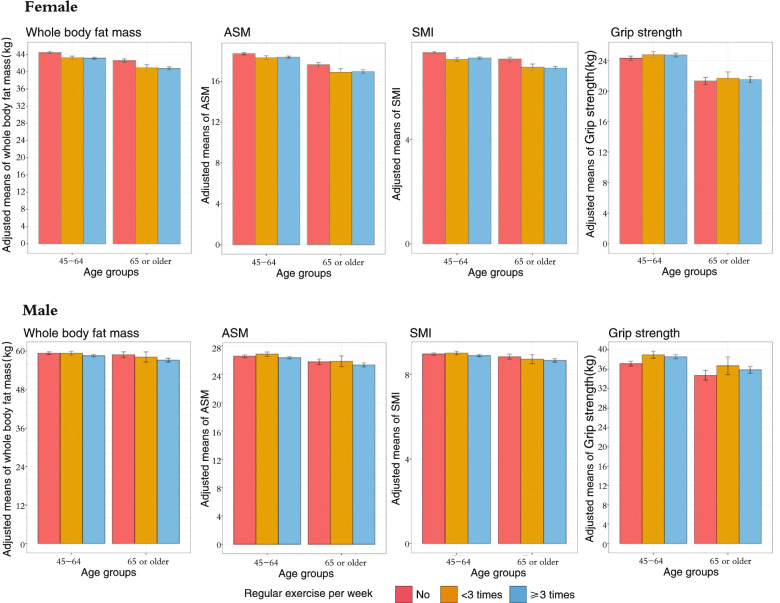
Adjusted means (standard error) of sarcopenia related traits across exercise and age groups. Age in years and race/ethnicity; plus menopause status for females were adjusted. ASM = appendicular skeletal muscle mass (the added muscle mass of four limbs). SMI = skeletal muscle index. *P* < 0.001 for all traits

The adjusted mean differences of obesity-related traits across gender and exercise groups are displayed in Figs. 1 and 2. Overall, after adjusting for age and race/ethnicity (plus post-menopause status in females), all obesity-related traits exhibited inversely liner trends across the three exercise categories. The *p*-values were less than 0.001 for all groups. On the contrary, the adjusted mean differences of sarcopenia-related traits across age and exercise groups varied. Overall, in middle-aged and older adults, the associations of higher exercise frequencies and sarcopenia-related traits exhibited mixed trends.

Table 2 shows the results of the generalized linear models assessing the associations of exercise frequency levels with obesity related traits after controlling for age, race/ethnicity, menopause status and lifestyle factors. In middle-aged females, exercising ≥ 3 times/week was significantly associated with lower BMI (b = -2.01, 95% CI = [-2.54, -1.47]), waist circumference (b = -5.70, 95% CI = [-5.21, -1.52]), fat mass (b = -4.38, 95% CI = [-5.05, -3.70]) and percent of body fat (b = -3.17, 95% CI = [-3.54, -2.79]). Similar results with less effects were reported in middle-aged females who regularly exercised one to two times per week, except lean mass. Similar results with higher effects were found in older females, with older females experiencing the highest reductions in almost all parameters. In males, regular exercise ≥ 3 times/week was significantly associated with lower BMI, waist circumference, fat mass, and percent of fat. However, exercise less than three times per week only showed negative association of percent body fat in middled males (b = 0.81, 95% CI = [-1.43, -0.19]).

**Table 2:** Linear regression models of physical activity with obesity related traits by gender and age groups

		**Female**					**Male**		
**Age (years)**	**Variables**	**Regular exercise- < 3/week b (95% CI)**		**Regular exercise- ≥ 3/week b (95% CI)**			**Regular exercise- < 3/week b (95% CI)**	**Regular exercise- ≥ 3/week b (95% CI)**	
		Reference = no regular exercise							
**45 to 64**	BMI	-1.51**(-2.34, -0.69)	-2.01**(-2.54, -1.47)			-0.42(-0.99, 0.14)			-0.94**(-1.29, -0.59)
	Waist circumference (cm)	-3.39***(-5.26, -1.52)	-5.70***(-5.21, -1.52)			-1.72(-3.92, 0.48)			-3.10***(-4.46, -1.74)
	Whole body fat mass (kg)	-2.41**(-3.45, -1.37)	-4.38**(-5.05, -3.70)			-0.83(-1.79, 0.13)			-1.99** (-2.59, -1.39)
	% Fat	-1.47**(-2.05, -0.89)	-3.17**(-3.54, -2.79)			-0.81**(-1.43, -0.19)			-1.41** (-1.75, -0.98)
** ≥ 65**		Reference = no regular exercise							
	BMI	-2.23**(-3.49, -0.96)			-2.96**(-3.73, -2.20)	-0.38 (-1.89, 1.13)			-1.89**(-2.74, -1.04)
	Waist circumference (cm)	-5.72(-9.02, -2.42)			-6.93***(-9.02, -2.42)	0.22(-4.85,5.28)			-5.59***(-8.42, -2.76)
	Whole body fat mass (kg)	-3.31**(-5.30. -1.32)			-4.55**(-5.76, -3.34)	-0.80 (-1.86, 3.45)			-3.36**(-4.84, -1.87)
	% Fat	-1.56**(-0.50, -0.35)			-2.66**(-0.61, -1.92)	0.92 (-0.83, 2.66)			-1.97**(-2.94, -0.99)

All models were adjusted for age in years, race/ethnicity, calcium supplement intake, smoking status, alcohol drinking, milk consumption, and at least 15 min sun exposure a day, plus postmenopausal in females

Note: BMI = Body mass index; Fat mass = whole body fat mass; % Fat = percent of body fat

Regular exercise: “no regular exercise” as the reference group; b = regression parameter estimates

* < 0.05. ** < 0.01 *** < 0.001

### Physical activity and sarcopenia related traits

Table 3 shows the results of the associations of exercise frequency levels with sarcopenia related traits after controlling for age, race/ethnicity, menopause status and lifestyle factors in model 1 s, with additional adjustment for weight in model 2 s. In middle-aged and older females, in model 1 s, compared to those who did not exercise regularly, middle-aged and older females who engaged ≥ 3 times/week had lower lean mass, ASM, SMI and comparable grip strength. However, after further controlling for body weight (model 2 s), both middle-aged and older females who exercised at least 3 times/week exhibited higher whole body lean mass, ASM, SMI and higher grip strength, except SMI (b = 0.05, 95%CI = [-0.17, 0.12] and grip strength (b = 0.63, 95%CI = (-0.32, 1.81]) in older females. For middle-aged males, any regular exercise was positively related to grip strength in all models. Positive associations between exercise frequency levels and other parameters were found only after further adjusting for weight.

**Table 3:** Linear regression models of physical activity with sarcopenia related traits by gender and age groups

		**Female**		**Male**		
**Age (years)**	**Variables**	**Regular exercise- < 3/week b (95% CI)**	**Regular exercise- ≥ 3/week b** **(95% CI)**	**Regular exercise- < 3/week** **b (95% CI)**		**Regular exercise-** ** ≥ 3/week** **b (95% CI)**
**45 to 64**	**Model 1**	Reference = no regular exercise		Reference = no regular exercise		
	Whole body lean mass	-0.90*(-1.63, -0.18)	-0.84**(-1.31, -0.37)	0.38 (-0.67, 1.42)		-0.59*(-1.24, 0.06)
	ASM	-0.39 (-0.77, 0.01)	-0.33** (-0.58, -0.08)	-0.27 (-0.63, 0.10)		0.17 (-0.43, 0.77)
	SMI	-0.23 (-0.37, -0.09)	-0.16*** (-0.11, -0.22)	-0.08 (-0.19, 0.02)		0.03 (-0.36, 0.20)
	Grip strength	0.18 (-0.61, 0.98)	0.26 (-0.25, 0.78)	1.36**(0.59, 2.13)		1.78**(0.54, 3.02)
	**Model 2**	Reference = no regular exercise		Reference = no regular exercise		
	Whole body lean mass	0.31 (-0.04, 0.66)	1.12***(0.89, 1.34)	0.65* (0.13, 1.56)		0.67***(0.35, 0.99)
	ASM	0.57 (0.28, 0.87)	0.46*** (0.27, 0.87)	0.23* (0.05, 0.43)		0.71*** (0.58, 0.83)
	SMI	-0.03 (-0.11, 0.49)	0.16***(0.10, 0.21)	0.13 (-0.10, 2.72)		0.10*** (0.82, 2.57)
	Grip strength	0.57 (-0.34, 1.47)	0.68**(0.11, 1.26)	1.31* (0.13, 2.94)		1.70**(0.93, 2.67)
** ≥ 65**	**Model 1**	Reference = no regular exercise			Reference = no regular exercise	
	Whole body lean mass	-1.99**(-3.38, -0.61)	-2.03**(-2.87, -1.19)	-0.54 (-3.41, 2.34)		-1.99*(-3.60, -0.39)
	ASM	-0.78***(-1.19, -0.37)	-0.96**(-1.63, -0.28)	0.38 (-1.39, 0.11)		0.17 (-1.18, 1.52)
	SMI	-0.36** (-0.37, -0.21)	-0.37*** (-0.11, -0.12)	-0.08 (-0.45, 0.30)		-0.22* (-0.43, -0.01)
	Grip strength	-0.10 (-1.66, 1.46)	0.10 (-0.84, 1.05)	2.11 (-1.57, 5.78)		0.62 (-1.44, 2.68)
	**Model 2**	Reference = no regular exercise			Reference = no regular exercise	
	Whole body lean mass	0.14 (-0.52, 1.81)	0.55**(0.14, 0.96)	-0.54 (-1.72, 0.63)		0.91**(0.23, 1.57)
	ASM	0.03 (-0.33, 0.39)	0.42*** (0.19, 0.64)	0.12 (-0.58, 0.83)		0.63** (0.23, 1.03)
	SMI	-0.03 (-0.04, 0.14)	0.05 (-0.17, 0.12)	-0.09 (-0.07, 0.23)		0.08 (-0.36, 0.17)
	Grip strength	0.27 (-0.27, 0.73)	0.63 (-0.32, 1.81)	2.42 (-1.57, 5.78)		1.90 (-0.09, 3.89)

In model 1, age in years, race/ethnicity, calcium supplement intake, smoking status, alcohol drinking, milk consumption, and at least 15 min sun exposure a day, plus postmenopausal in females. In model 2, in addition to variables adjusted in model 1, body weight was adjusted

Note: BMI = Body mass index; Lean mass = whole body lean mass; ASM = appendicular skeletal muscle mass (the added muscle mass of four limbs). SMI = skeletal muscle index. Regular exercise: “no regular exercise” as the reference group; b = regression parameter estimates

* < 0.05. ** < 0.01 *** < 0.001

### Physical activity and body composition status

**Fig. 3:**
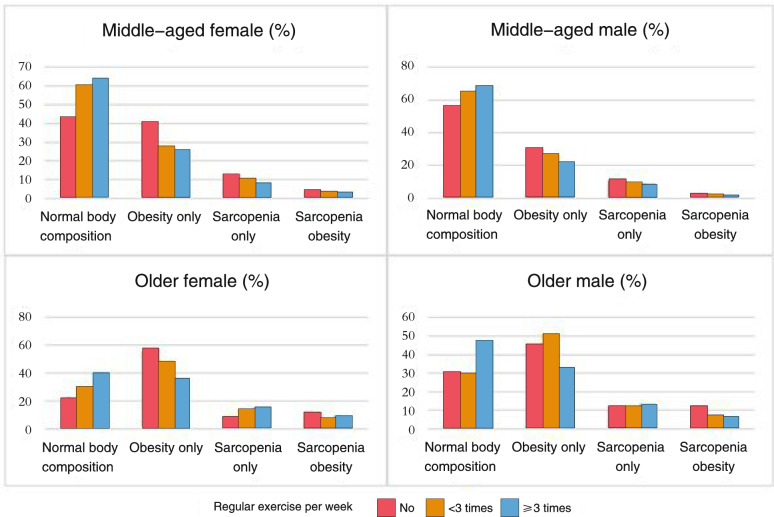
The distribution of normal body composition, obesity, sarcopenia, and obesity sarcopenia by physical activity, gender and age groups. *P* < 0.001 for all groups (except p < 0.01 for older male)

Figure 3 shows the distribution of the four categories of body composition status across exercise frequency levels. Overall, those with higher exercise frequencies per week exhibited higher proportions of individuals with normal body composition and lower proportions of people with obesity (*p* < 0.01 for older male group and *p* < 0.001 for all other groups). Higher exercise frequencies were associated with lower trends of sarcopenia and sarcopenic obesity in middle-aged adults, but a higher trends of sarcopenia prevalence in older adults. Table 4 displays the ordinal logistic regression results of body composition status by gender and age groups. In both middle-aged females and males, those exercising ≥ 3 times/week were less like to have obesity, sarcopenia and sarcopenic obesity comparing to those without regular exercise. The findings sustained for those regular exercised 1–2 times/week, except for having sarcopenia (odds = 0.77, 95% = [0.52, 1.14] for females; and odds = 0.75, 95%CI = [0.51, 1.09] for males). In both older females and males, only those exercising ≥ 3 times/week were less like to have obesity and sarcopenic obesity comparing to those without regular exercise. No differences were found between exercise frequency levels and sarcopenia. Regular exercising 1 to 2 times per week yield no favorable results in all conditions in older adults.

**Table 4:** Ordinal logistic regression modes of body composition status by gender and age groups

		**Female**		**Male**	
**Age (years)**	**Body composition status**	**Regular exercise- < 3/week** **Odds (95% CI)**	**Regular exercise-** ** ≥ 3/week** **Odds (95% CI)**	**Regular exercise- < 3/week** **Odds (95% CI)**	**Regular exercise-** ** ≥ 3/week** **Odds (95% CI)**
**45 to 64**	Reference = normal body composition	Reference = no regular exercise		Reference = no regular exercise	
	Obesity only	0.66***(0.55, 0.79)	0.40***(0.33, 0.47)	0.64**(0.48, 0.86)	0.66***(0.55, 0.79)
	Sarcopenia only	0.77 (0.52, 1.14)	0.76* (0.59, 0.98)	0.75 (0.51, 1.09)	0.71* (0.56, 0.79)
	Sarcopenic obesity	0.50**(0.33, 0.47)	0.32*(0.24, 0.42)	0.43*(0.21, 0.86)	0.56*(0.39, 0.80)
** ≥ 65**	Reference = normal body composition	Reference = no regular exercise		Reference = no regular exercise	
	Obesity only	0.64 (0.37, 1.09)	0.39***(0.28, 0.54)	1.36 (0.60, 3.12)	0.49** (0.31, 0.78)
	Sarcopenia only	1.36 (0.64, 2.92)	1.04 (0.63, 1.71)	1.01 (0.30, 3.38)	0.84 (0.43, 1.62)
	Sarcopenic obesity	0.55 (0.24, 1.29)	0.52**(0.32, 0.85)	0.63 (0.15, 2.58)	0.38*(0.18, 0.80)

Models were adjusted for age in years, race/ethnicity, calcium supplement intake, smoking status, alcohol drinking, milk consumption, and at least 15 min sun exposure a day, plus postmenopausal in females

Regular exercise: “no regular exercise” as the reference group; Odds = Odds ratio

* < 0.05. ** < 0.01 *** < 0.001

Note: Obesity was defined as met one or more of the following criteria: 1) BMI ≥ 30, 2) % Fat (> 42 for female, > 30 for male), or 3) waist circumference (≥ 88 cm for female, ≥ 102 cm for male). Sarcopenia was defined as grip strength < 16 kg and skeletal muscle index < 5.5 for female, and grip strength < 27 kg and skeletal muscle index < 7 for male

## Discussion

This cross-sectional study examined the associations of physical activity levels with obesity, sarcopenia, and sarcopenic obesity in a large and diverse population. Overall, the beneficial associations were found for obesity and obesity related traits in both middle-aged and older adults, especially those exercising ≥ 3 times/week. However, sarcopenia related traits, were negatively related to higher exercise frequencies, and favorable sarcopenia related traits with exercise frequencies were only found after adjusting for body weight. Additionally, in both older females and males, only those exercising ≥ 3 times/week were less like to have obesity and sarcopenic obesity comparing to those without regular exercise, and regular exercising 1 to 2 times/week was not favorably related to any conditions. Exercising 1 to 2 times/week in middle-aged adults and any regular exercise in older adults were not related to less sarcopenia. The study findings may suggest that physical activity and exercise regimens to balance obesity and sarcopenia related traits should be promoted in older adults. The complex associations among physical activity, weight reduction and muscle maintaining need to be further explored in the aging population to maximize the benefits of exercise.

Compared to people who did not engage in regular exercise, those engaged in regular exercise ≥ 3 times/week had lower BMI, fat mass, waist circumference and percent body fat across age and gender groups; the associations were less or not significant in some body composition indicators if exercise was practiced less than 3 times per week. The findings are consistent with a widely accepted evidence exercise can decrease BMI and fat mass [46, 47]. The percent body fat for males (23.45%-25.80%) and females (34.81%-39.01%) found in this study were similar to the findings using the national representative data the National Health and Nutrition Examination Survey (NHANES) 1999–2004 [48]. Given BMI, fat mass and percent of body fat are closely related to a numerous metabolic and disease outcome [49, 50], our study findings affirm that regular exercise might be an effective way to improve health outcomes, and exercise three times per week may increase the benefits.

However, relationship between exercise frequency and lean mass varied across age and gender groups in the current study. Higher exercise frequencies were negatively related to lean mass in middle-aged females and both older males and females, which was attenuated after considering for body weight. Muscle mass is essential in preventing some of the most common and increasingly prevalent health conditions (e.g., obesity, diabetes, sarcopenia) throughout one’s life span [51, 52]. However, muscle mass begins to decline approximately 3–8% every decade starting around the age of 30, and the decline is faster after the age of 60 [53]. Exercise, especially resistance training, has been evidenced to be an effective approach in increasing muscle mass [51, 54]. First, the current findings may suggest the current exercise practice (e.g., type, intensity) is not optimal and holistic for health benefits in middle-aged and older adults in the community. This is consistent with previous reports resistance exercise training might be difficult to implement, especially in community-dwelling older individuals, partially due to the need of specific equipment and supervision, and the possibility that resistance exercise is not stressed in certain conditions frequently found in older patients (e.g., hypertension, diabetes, stroke) [51]. Additionally, energy restriction along with more exercise was reported to be related to muscle reduction [55]. Dietary data was not available in this study. Furthermore, the results may also reflect each individual responds differently to physical activity given various variations in genes, lifestyle behavior, age, gender, and genetic-environmental interaction. Therefore, a personalized lifestyle intervention may yield the best muscle mass outcomes in the aging population [56, 57].

Interestingly, after adjusting for body weight, the negative associations of exercise frequency with muscle mass become positive or neutral. Meanwhile, we found body weight was negatively related to exercise levels in all age and gender groups and positively related to all sarcopenia-related traits in all groups (not shown in tables). Due to the cross-sectional nature of the study, a causal relationship between exercise frequency and body composition traits could not be established in the current study. Previous evidence show that increased adiposity could be a chronic physical overload stimulus on the antigravity muscles, such as quadriceps and calf, thus increasing muscle size and strength [58]. In addition, it is reported that weight loss could accelerate sarcopenia in older adults [44]. Therefore, it’s possible that the exercise induced weight loss may consequently reduce the effect of physical loading of body weight on lean mass, especially when accompanied with diet restriction or inappropriate diet selection. Meanwhile, significant muscle mass increase could be achievable only with progressive resistance training or weight gain through extra energy and protein intake [37]. Therefore, maintenance of muscle mass might have been overlooked or under emphasized for weight loss purpose in middle-aged and older adults in current practice, with the norm that obesity is a major public health emergency and crisis [59]. Physical activity and exercise programs to balance body composition and maximize health outcomes need to be holistically explored.

The study also found higher frequency of exercise was positively related to grip strength in middle-aged males after controlling for included covariates. The similar findings were found in middle-aged females only after controlling for body weight; but no associations were found in older adults. The physical activity effect on grip strength in older adults is inconsistent from current literature [60, 61]. But exercise intervention with more emphasis on resistance training involving multiple body parts and higher weekly frequency reported grip strength increase comparing to control group [61]. The current study, with a cross-sectional nature, found no relationships between higher frequency of exercise and grip strength in older adults may suggest that the exercise type might have been overlooked in the general older population.

Lastly, in both older males and females, exercising less than 3 times/week was not related to less likelihood to have obesity or sarcopenic obesity, and any exercise frequencies was not related to less odds of sarcopenia. The findings partially conflict with previous conclusions that physical activity is negatively associated to sarcopenia [20]. Some possible reasons are that: 1) the previous studies didn’t differentiate sarcopenia and sarcopenic obesity, so different results might be found [20, 62], or the studies were conducted in other countries [22, 23]. In addition, the EWGSOP2 revised the definition of sarcopenia with the aim to promote early detection and treatment, and we used this most updated definition for sarcopenia [33], the results generated from which might be different from previous studies. Furthmore, the current findings may also suggest that exercise regimens to maintain and improve muscle mass and strength are needed in older adults, and older adults with sarcopenia may not engage in regular exercise.

## Limitations

There are several limitations of this study. First, physical activity assessed using exercise frequency was self-reported, indicating a probability of recall bias. Second, we only assessed regular exercise frequency per week, which might not capture all forms of physical activity. However, it is evidenced that weekly exercise frequency is essential for health benefits [63, 64]. Third, the intensity, duration and specific exercise type were not assessed. Fifth, despite we included various covariates in the models, other factors (e.g., dietary intake, sedentary behavior, sleep patterns, medication use) [65–68], which are related to body composition, were not assessed in the current study. Particularly, dietary intake (e.g., protein) is essential to lean muscle mass [66], but only calcium rich food consumption was examined in the current study. Sixth, given the cross-sectional nature of the study, causality associations cannot be generated. Lastly, even though the study participants are racially and ethnically diverse, they were enrolled in one region in the southern United States, therefore the generalizability to other populations may be limited.

## Conclusion

Despite some limitations, using the data from a large and diverse cohort this study confirms that physical activity is associated with favorable obesity- and sarcopenia-related traits. Higher regular exercise frequencies are negatively associated with BMI, fat mass, and percent body fat in all age and gender groups. However, higher regular exercise frequencies were also negatively related to lean mass and showed no associations to grip strength in older adults before adjusting for body weight. Similarly, exercise 1–2 times/week in middle-aged adults and any exercise frequencies in older adults were not associated with less odds of having sarcopenia. Recognizing the relationships between exercise frequency and muscle mass and strength outcomes across different age and gender groups may not only reflect the complex physiological mechanisms, but also informs the intervention gaps. Balancing body composition (e.g., reducing fat mass and weight while maintaining muscle mass) to maximize health outcomes holistically needs to be explored, especially in middle-aged and older adults. The findings of this study may suggest individualized physical activity regimens which increase or preserve lean mass and grip strength need to be further emphasized, especially in older adults to improve healthy aging.

## Data Availability

The datasets used in the current study are not publicly available due to the study is still on going, but are available from the corresponding author on reasonable request.

## Abbreviations

ASM: Appendicular skeletal lean mass
BMI: Body mass index
CI: Confidence interval
DXA: Dual-energy X-ray absorptiometry
DXA: European Working Group on Sarcopenia in Older People
LOS: Louisiana Osteoporosis Study
NHANES: National Health and Nutrition Examination Survey
ORs: Odds ratio
SMI: Skeletal muscle index

## References

[1] Porter, J.L. and M. Varacallo, Osteoporosis, in StatPearls. 2020, StatPearls Publishing Copyright © 2020, StatPearls Publishing LLC.: Treasure Island (FL).

[2] Cannarella R, et al. Osteoporosis from an Endocrine Perspective: The Role of Hormonal Changes in the Elderly. J Clin Med. 2019;8(10):1564. 10.3390/jcm8101564 PMC6832998 31581477

[3] He C (2020). Bone and Muscle Crosstalk in Aging. Front Cell Dev Biol.

[4] Buckinx F, Aubertin-Leheudre M (2019). Relevance to assess and preserve muscle strength in aging field. Prog Neuropsychopharmacol Biol Psychiatry.

[5] Millar B (2020). Investigating musculoskeletal health and wellbeing; a cohort study protocol. BMC Musculoskelet Disord.

[6] Xu BY (2019). Predictors of poor functional outcomes and mortality in patients with hip fracture: a systematic review. BMC Musculoskelet Disord.

[7] Kirk B, Zanker J, Duque G (2020). Osteosarcopenia: epidemiology, diagnosis, and treatment-facts and numbers. J Cachexia Sarcopenia Muscle.

[8] Zhou Y (2019). Geographical differences in osteoporosis, obesity, and sarcopenia related traits in white American cohorts. Sci Rep.

[9] Westerterp KR (2018). Exercise, energy balance and body composition. Eur J Clin Nutr.

[10] U.S. Department of Health and Human Services. Physical activity guidelines for Americans, 2nd 2018 [cited 2021 May 1]; Available from: https://health.gov/sites/default/files/2019-09/Physical_Activity_Guidelines_2nd_edition.pdf.

[11] Slattery M (1992). Associations of body-fat and its distribution with dietary-intake, physical-activity, alcohol, and smoking in blacks and whites. Am J Clin Nutr.

[12] Trudelle-Jackson, E., A.W. Jackson, and J.R. Morrow, Jr., Relations of meeting national public health recommendations for muscular strengthening activities with strength, body composition, and obesity: the Women's Injury Study.(RESEARCH AND PRACTICE)(Author abstract)(Report)*.* Am J Public Health, 2011. 101(10): p. 1930. 10.2105/AJPH.2011.300175 PMC3174351 21852647

[13] Ramage S (2014). Healthy strategies for successful weight loss and weight maintenance: a systematic review. Appl Physiol Nutr Metab.

[14] Stoner L (2016). Efficacy of Exercise Intervention for Weight Loss in Overweight and Obese Adolescents: Meta-Analysis and Implications. Sports Med.

[15] Speakman JR, Westerterp KR (2010). Associations between energy demands, physical activity, and body composition in adult humans between 18 and 96 y of age. Am J Clin Nutr.

[16] Villareal DT (2017). Aerobic or Resistance Exercise, or Both, in Dieting Obese Older Adults. N Engl J Med.

[17] Westerterp KR (2018). Changes in physical activity over the lifespan: impact on body composition and sarcopenic obesity. Obes Rev.

[18] Labott BK (2019). Effects of Exercise Training on Handgrip Strength in Older Adults: A Meta-Analytical Review. Gerontology.

[19] Oliveira JS (2020). Evidence on Physical Activity and the Prevention of Frailty and Sarcopenia Among Older People: A Systematic Review to Inform the World Health Organization Physical Activity Guidelines. J Phys Act Health.

[20] Steffl M (2017). Relationship between sarcopenia and physical activity in older people: a systematic review and meta-analysis. Clin Interv Aging.

[21] Zeng P (2016). Sarcopenia-related features and factors associated with lower muscle strength and physical performance in older Chinese: a cross sectional study. BMC Geriatr.

[22] Aggio DA (2016). Cross-sectional associations of objectively measured physical activity and sedentary time with sarcopenia and sarcopenic obesity in older men. Prev Med.

[23] Ryu M (2013). Association of physical activity with sarcopenia and sarcopenic obesity in community-dwelling older adults: the Fourth Korea National Health and Nutrition Examination Survey. Age Ageing.

[24] Kim SH, Kim TH, Hwang HJ (2013). The relationship of physical activity (PA) and walking with sarcopenia in Korean males aged 60 years and older using the Fourth Korean National Health and Nutrition Examination Survey (KNHANES IV-2, 3), 2008–2009. Arch Gerontol Geriatr.

[25] Park HY (2021). Relationship Between Sarcopenia, Obesity, Osteoporosis, and Cardiometabolic Health Conditions and Physical Activity Levels in Korean Older Adults. Front Physiol.

[26] Veen J, et al. Engagement in Muscle-Strengthening Activities Lowers Sarcopenia Risk in Older Adults Already Adhering to the Aerobic Physical Activity Guidelines*. *Int J Environ Res Public Health. 2021;18(3):989. 10.3390/ijerph18030989 PMC7908493 33499423

[27] von Berens Å (2020). Sarcopenic obesity and associations with mortality in older women and men - a prospective observational study. BMC Geriatr.

[28] Volpi E, Nazemi R, Fujita S (2004). Muscle tissue changes with aging. Curr Opin Clin Nutr Metab Care.

[29] Watanabe D (2021). Factors associated with sarcopenia screened by finger-circle test among middle-aged and older adults: a population-based multisite cross-sectional survey in Japan. BMC Public Health.

[30] Du Y (2017). Socioeconomic status and bone mineral density in adults by race/ethnicity and gender: the Louisiana osteoporosis study. Osteoporos Int.

[31] Ning HT (2021). Racial and gender differences in the relationship between sarcopenia and bone mineral density among older adults. Osteoporos Int.

[32] Beaudart C (2016). Sarcopenia in daily practice: assessment and management. BMC Geriatr.

[33] Cruz-Jentoft AJ (2019). Sarcopenia: revised European consensus on definition and diagnosis. Age Ageing.

[34] Zhao Q (2018). Metabolomic profiles associated with bone mineral density in US Caucasian women. Nutr Metab (Lond).

[35] O'Donovan G (2017). Association of "Weekend Warrior" and Other Leisure Time Physical Activity Patterns With Risks for All-Cause, Cardiovascular Disease, and Cancer Mortality. JAMA Intern Med.

[36] Stofan JR (1998). Physical activity patterns associated with cardiorespiratory fitness and reduced mortality: the Aerobics Center Longitudinal Study. Am J Public Health.

[37] Izquierdo M (2021). International Exercise Recommendations in Older Adults (ICFSR): Expert Consensus Guidelines. J Nutr Health Aging.

[38] Fleury N, Geldenhuys S, Gorman S. Sun Exposure and Its Effects on Human Health: Mechanisms through Which Sun Exposure Could Reduce the Risk of Developing Obesity and Cardiometabolic Dysfunction. Int J Environ Res Public Health. 2016;13(10):999. 10.3390/ijerph13100999 PMC5086738 27727191

[39] Graff-Iversen S (2019). Associations of tobacco smoking with body mass distribution; a population-based study of 65,875 men and women in midlife. BMC Public Health.

[40] Liangpunsakul S, Crabb DW, Qi R (2010). Relationship among alcohol intake, body fat, and physical activity: a population-based study. Ann Epidemiol.

[41] Radavelli-Bagatini S (2013). Association of dairy intake with body composition and physical function in older community-dwelling women. J Acad Nutr Diet.

[42] Kastner M (2006). Age-specific search strategies for Medline. J Med Internet Res.

[43] Seimon RV (2019). Effect of Weight Loss via Severe vs Moderate Energy Restriction on Lean Mass and Body Composition Among Postmenopausal Women With Obesity: The TEMPO Diet Randomized Clinical Trial. JAMA Netw Open.

[44] Newman, A.B., et al., Weight change and the conservation of lean mass in old age: the Health, Aging and Body Composition Study*.* Am J Clin Nutr, 2005. 82(4): p. 872–8; quiz 915–6. 10.1093/ajcn/82.4.872 16210719

[45] Johnston R, Jones K, Manley D (2018). Confounding and collinearity in regression analysis: a cautionary tale and an alternative procedure, illustrated by studies of British voting behaviour. Qual Quant.

[46] Barrow DR (2019). Exercise prescription for weight management in obese adults at risk for osteoarthritis: synthesis from a systematic review. BMC Musculoskelet Disord.

[47] Kim KB (2019). Effects of Exercise on the Body Composition and Lipid Profile of Individuals with Obesity: A Systematic Review and Meta-Analysis. J Obes Metab Syndr.

[48] Borrud, L.G., et al., Body composition data for individuals 8 years of age and older: U.S. population, 1999–2004*.* Vital Health Stat 11, 2010(250): p. 1–87. PMC5901781 20812448

[49] Ramírez-Vélez R, et al. Percentage of Body Fat and Fat Mass Index as a Screening Tool for Metabolic Syndrome Prediction in Colombian University Students*.* Nutrients. 2018;9(9):1009. 10.3390/nu9091009 PMC5622769 28902162

[50] Aune D (2017). Body mass index, abdominal fatness, fat mass and the risk of atrial fibrillation: a systematic review and dose-response meta-analysis of prospective studies. Eur J Epidemiol.

[51] Wolfe RR (2006). The underappreciated role of muscle in health and disease. Am J Clin Nutr.

[52] Woods R (2020). Association of lean body mass to menopausal symptoms: The Study of Women's Health Across the Nation. Womens Midlife Health.

[53] Pereira AF (2013). Muscle tissue changes with aging. Acta Med Port.

[54] Burrup R (2018). Strength training and body composition in middle-age women. J Sports Med Phys Fitness.

[55] Stokes, T., et al., *Recent Perspectives Regarding the Role of Dietary Protein for the Promotion of Muscle Hypertrophy with Resistance Exercise Training.* Nutrients, 2018. 10(2). 10.3390/nu10020180 PMC5852756 29414855

[56] Buford TW, Roberts MD, Church TS (2013). Toward exercise as personalized medicine. Sports Med.

[57] Peterson MD, Sen A, Gordon PM (2011). Influence of resistance exercise on lean body mass in aging adults: a meta-analysis. Med Sci Sports Exerc.

[58] Tomlinson DJ (2016). The impact of obesity on skeletal muscle strength and structure through adolescence to old age. Biogerontology.

[59] Ayton A, Ibrahim A (2019). Obesity is a public health emergency. BMJ.

[60] Santanasto AJ (2017). Effect of Physical Activity versus Health Education on Physical Function, Grip Strength and Mobility. J Am Geriatr Soc.

[61] Yoon DH, Lee JY, Song W (2018). Effects of Resistance Exercise Training on Cognitive Function and Physical Performance in Cognitive Frailty: A Randomized Controlled Trial. J Nutr Health Aging.

[62] Meier NF, Lee DC (2020). Physical activity and sarcopenia in older adults. Aging Clin Exp Res.

[63] Kemmler W, von Stengel S (2014). Dose-response effect of exercise frequency on bone mineral density in post-menopausal, osteopenic women. Scand J Med Sci Sports.

[64] Powell KE, et al. The Scientific Foundation for the Physical Activity Guidelines for Americans, 2nd Edition. J Phys Act Health. 2018:1–11. 10.1123/jpah.2018-0618 30558473

[65] Corona G (2016). Testosterone supplementation and body composition: results from a meta-analysis of observational studies. J Endocrinol Invest.

[66] Kim JE (2016). Effects of dietary protein intake on body composition changes after weight loss in older adults: a systematic review and meta-analysis. Nutr Rev.

[67] Saunders, T.J., et al., Sedentary behaviour and health in adults: an overview of systematic reviews*.* Appl Physiol Nutr Metab, 2020. 45(10 (Suppl. 2)): p. S197-s217. 10.1139/apnm-2020-0272 33054341

[68] Thomas EA, et al. Later Meal and Sleep Timing Predicts Higher Percent Body Fat. Nutrients. 2020;13(1):73. 10.3390/nu13010073 PMC7823810 33383648

